# Early Identification of Chronic Lung Allograft Dysfunction: The Need of Biomarkers

**DOI:** 10.3389/fimmu.2019.01681

**Published:** 2019-07-17

**Authors:** Adrien Tissot, Richard Danger, Johanna Claustre, Antoine Magnan, Sophie Brouard

**Affiliations:** ^1^Centre de Recherche en Transplantation et Immunologie (CRTI), INSERM, Université de Nantes, Nantes, France; ^2^Service de Pneumologie, Institut du Thorax, CHU Nantes, Nantes, France; ^3^Institut de Transplantation Urologie Néphrologie (ITUN), CHU Nantes, Nantes, France; ^4^Service Hospitalo-Universitaire de Pneumologie – Physiologie, CHU Grenoble Alpes, Grenoble, France; ^5^UMR S 1087 CNRS UMR 6291, Institut du Thorax, CHU Nantes, Université de Nantes, Nantes, France

**Keywords:** lung transplantation, chronic lung allograft dysfunction, BOS, RAS, biomarker, blood, bronchoalveolar lavage fluid

## Abstract

A growing number of patients with end-stage lung disease have benefited from lung transplantation (LT). Improvements in organ procurement, surgical techniques and intensive care management have greatly increased short-term graft survival. However, long-term outcomes remain limited, mainly due to the onset of chronic lung allograft dysfunction (CLAD), whose diagnosis is based on permanent loss of lung function after the development of irreversible lung lesions. CLAD is associated with high mortality and morbidity, and its exact physiopathology is still only partially understood. Many researchers and clinicians have searched for CLAD biomarkers to improve diagnosis, to refine the phenotypes associated with differential prognosis and to identify early biological processes that lead to CLAD to enable an early intervention that could modify the inevitable degradation of respiratory function. Donor-specific antibodies are currently the only biomarkers used in routine clinical practice, and their significance for accurately predicting CLAD is still debated. We describe here significant studies that have highlighted potential candidates for reliable and non-invasive biomarkers of CLAD in the fields of imaging and functional monitoring, humoral immunity, cell-mediated immunity, allograft injury, airway remodeling and gene expression. Such biomarkers would improve CLAD prediction and allow differential LT management regarding CLAD risk.

## Introduction

The number of patients who undergo lung transplantation (LT) has been rising steadily since the first LT era in the late 1980s. More than 4,000 procedures are performed each year worldwide (1). The growing interest in and commitment to this highly complex and problematic field of solid organ transplantation is due to global improvements in LT outcomes and the recent broadening of the donor pool. In the early days of LT, the mortality rate at 1 year post-transplant reached 30%, and several teams recently reported >90% survival at 12 months (2, 3). Moreover, the implementation of policies for marginal donor use has greatly increased the number of lungs available for patients with end-stage respiratory failure. This advancement was made possible by two factors. First, initial studies of marginal donor lungs reported short-term outcomes similar to those of so-called ideal donor lungs. Second, *ex vivo* lung perfusion techniques can be used to improve and evaluate donor lungs with suboptimal oxygenation capacity caused by a reversible etiology. However, long-term outcomes remain poor compared to those of other types of solid organ transplantation, and the main limitation is chronic lung allograft dysfunction (CLAD), which affects around half of the patients at 5 years post-transplantation and is responsible for reduced survival, reduced quality of life and increased medico-economic costs (1, 4–6). Interestingly, after the first-year post-LT, the slopes of the survival curves are fairly similar between LTs performed in the early decades of the procedure (1990–1998 and 1999–2008) and LTs performed in recent years (2009–2015); this observation highlights the lack of an efficient therapeutic approach for preventing or reversing the CLAD process.

While no treatment is currently available to reverse CLAD after diagnosis, early identification of CLAD would allow proactive and targeted strategies to reverse the progression of the disease before irreversible allograft damage occurs. Inversely, the management of recipients identified as having a low risk of developing CLAD could be modified, with a reduction of immunosuppressive treatment or medical appointments, potentially improving health care efficacy, and quality of life.

For such risk stratification, there is a need for reliable biomarkers that potentially result from a combination of parameters that can diagnose CLAD early or predict CLAD development. In this review, we mainly focus on biomarkers from peripheral blood and bronchoalveolar lavage fluid (BALF). These compartments are defined as minimally invasive according the biomarker working group (7), in contrast to biopsy, which remains invasive and cannot be performed repeatedly for routine monitoring. However, no compartment is ideal: on one hand, BALF is performed during bronchoscopy and therefore cannot be considered as non-invasive. Its use is also hampered by the volume of solution used for irrigation, the dilution of the measured factors and the need for standardization. On the other hand, specific signals in the blood may be difficult to measure as homeostatic/systemic signal can interfere with specific allograft biomarkers.

To prepare this review, the terms “lung transplantation,” “chronic lung allograft dysfunction,” “CLAD,” “bronchiolitis obliterans,” “BOS,” “restrictive allograft syndrome,” “RAS,” “gene expression,” “antibody,” “HLA,” “cells,” or “biomarkers” were used alone and in combination to search in PubMed over the past 20 years. The most relevant and appropriate articles were then hand selected. According to few numbers of large studies, no selection based on sample size was performed. Each included article was read and reviewed independently by 2 authors.

## CLAD Definition and Diagnosis in Clinical Practice

CLAD is a global term, introduced in the past few years, encompassing all forms of chronic pulmonary function decline, after eliminating known causes (persistent acute rejection, infection, anastomotic stricture, or disease recurrence, pleural disease, diaphragm dysfunction or native lung hyperinflation) (8). Therefore, it is a heterogeneous entity in which two main phenotypes are currently identified. Bronchiolitis obliterans syndrome (BOS), defined by a persistent decline in the forced expiratory volume in 1 s (FEV1) and an obstructive functional pattern, was long thought to be the only manifestation of CLAD. Restrictive allograft syndrome (RAS) is now identified and characterized by persistent declines in forced vital capacity and total lung capacity associated with radiologic fibrotic changes. RAS presents the worst allograft survival, with median allograft survival of 4 years and <2 years after onset in patients with BOS and RAS, respectively (7–9).

**Figure 1:**
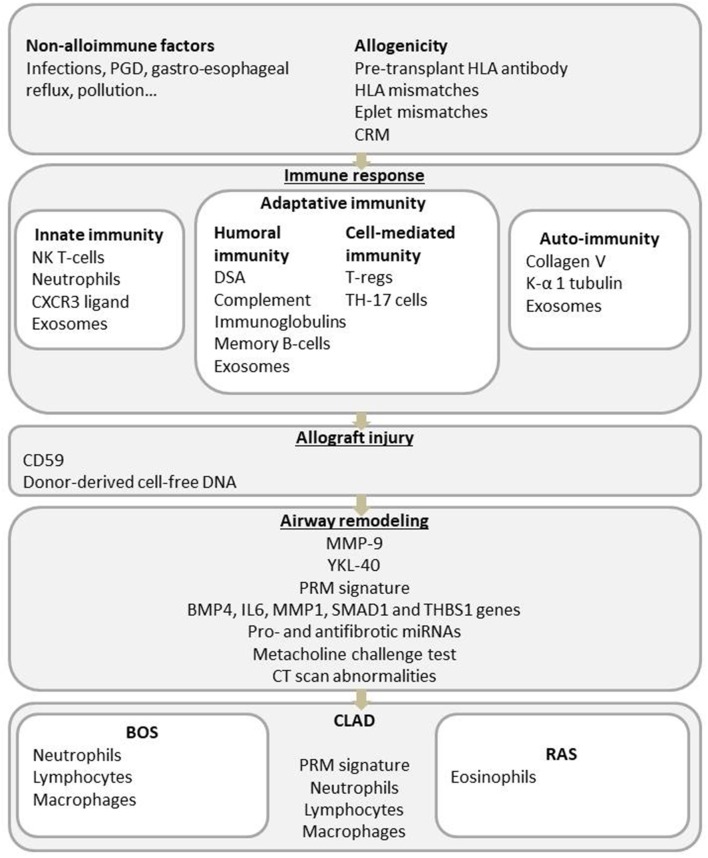
Main physiopathologic mechanisms involved in CLAD, with associated potential biomarkers.

The exact physiopathology of CLAD remains partially understood, but an increasing number of publications in the past decade have provided several clues about the dysfunctional process. Briefly, immunological risk factors (acute cellular rejection, lymphocytic bronchiolitis, self-antigen exposure, and donor-specific antibodies) and non-immune mechanisms (primary graft dysfunction (PGD), gastroesophageal reflux, bacterial or viral infections and pollutant exposure) lead to the activation of the innate and adaptive immune systems and chronic lung inflammation. Impaired wound healing and fibrosis of the lung will then end in small airway obstruction for the BOS phenotype or in parenchymal and pleural fibrosis for the restrictive CLAD phenotype (Figure 1) (10).

Since BOS accounts for the majority of CLAD cases, approximately 50% at 5 years post-LT, most studies describing CLAD-associated parameters are mainly BOS-centered.

## Imaging and Functional Monitoring

Precise evaluation of small airway pathology is extremely difficult, whether by functional outcomes, routine radiologic imaging techniques or transbronchial biopsy. Parametric response mapping (PRM) is a voxel-based methodology for image analysis that has been shown to provide a quantitative analysis of functional small airway diseases in the lung by comparing expiratory and inspiratory images from a chest computed tomography scanner (CT scan). Belloli et al. investigated the PRM signature of lung transplant recipients (LTRs) with persistent spirometric decline (22 patients) compared to that of LTR controls with no functional graft decline (11). This technique was able to efficiently discriminate patients with BOS and patients with RAS. The study also evaluated the prognostic capacity of the PRM value at the onset of spirometric decline in a retrospective cohort of 52 patients. The authors demonstrated that the degree of functional small airway disease assessed by PRM is a robust predictor of survival, with significantly poorer survival in patients with PRM values ≥30%. Moreover, this technique clearly has the potential to identify small airway disease earlier than pulmonary function tests, as some patients had a definite small airway disease pattern up to 1 year before the diagnosis of BOS. The Leuven group reported persistent chest CT scan abnormalities (small nodules, reticulations, peripheral patchy consolidations, and ground glass attenuation) that appeared almost 10 months before the clinical diagnosis of RAS (12). A radiological analysis of more than 300 CT scans from 22 patients showed two main distribution patterns: upper lobe fibrotic changes with upper lobe volume loss progressing in a multistep pattern of exacerbation or diffuse abnormalities with sudden onset and rapid progression. Patients with initial diffuse lesions had a worse prognosis than patients in the other group, with a graft survival difference of 27 months after the first onset of non-regressing CT scan lesions. Again, in the first pattern, the first non-regressing lung lesions appeared more than 13 months before the diagnosis of RAS, and for the more rapidly progressive pattern, the first non-regressing lung lesions appeared 3 months before CLAD diagnosis These studies suggest the usefulness of clinical imaging for early CLAD diagnosis and for BOS/RAS discrimination.

As for functional analysis, a recent retrospective study reported a promising use of methacholine challenge test (MCT) to predict CLAD. Bronchial hyperresponsiveness assessed by MCT in 140 LTR at 3 months post-transplantation was shown to be associated with the development of CLAD up to 3 years post-transplantation (13). The possibility to repeat MCT during LTR follow-up indicates an interesting predictive biomarker for immune monitoring.

## Allo-immunity Monitoring

### HLA Antigen Matching

Unlike to renal transplantation, there is less evidence of the impact of HLA mismatches on CLAD in lung transplantation. Some studies showed conflicting results (14, 15), but more recently, increasing data suggested that HLA mismatches are correlated with a higher risk of BOS (16, 17) and CLAD (18), either for class I mismatches (16, 17, 19) or for both class I and class II mismatches (20, 21). If HLA matching seems to be of interest in evaluating the risk of CLAD, that is mainly because HLA mismatches are associated with the development of anti-HLA donor-specific antibodies (DSAs) discussed below.

### Stereochemical Modeling of HLA Matching

The binding affinity between HLA antibodies and HLA antigens can be further analyzed by stereochemical modeling of the key antigen–antibody amino acid combinations. “Eplets” are short strings of amino acid residues within an epitope that directly influences antibody affinity. Using the theoretical computer algorithm *HLAMatchmaker*, Walton et al. characterized HLA matching between the recipient and the donor graft at the allele and eplet levels in a cohort of 175 patients from Albert Hospital in Australia (22). The Cox proportional hazard model was used to measure the influence of HLA mismatch on the hazards of CLAD, BOS, and RAS. HLA-A/B antigen and eplet mismatch were not independent predictors of these clinical events. Conversely, at the eplet level, HLA DR 1/3/4/5 DQ A/B mismatches were associated with the development of CLAD. For BOS analysis, only the HLA DQ A/B eplet mismatch showed a significant relationship with BOS, whereas all HLA Class II eplet mismatches were associated with RAS. Even if eplet matching offers a more specific indicator for recipient-donor pairing, it remains difficult to consider eplet matching as a limiting factor for the choice of recipient for a proposed donor allograft due to organ donation shortage. However, eplet matching for allograft allocation refinement could enable better personalized risk stratification for CLAD.

### HLA Polymorphisms and Genetic Variants as CLAD Risk Factors

Recent work from the Marseille group focused on the genetic polymorphism of non-classical HLA class I molecules (23, 24). HLA-G is a non-classical HLA class I molecule, with immune-tolerogenic properties. The level of soluble HLA-G in bodily fluids is suspected to correlate with clinical outcomes. The structure of the HLA-G haplotype can influence its expression and can be used to reliably predict soluble HLA-G levels. HLA-G was evaluated in the context of LT and CLAD. In a population of 138 LT recipients, the authors found that compared to patients with other HLA-G haplotypes, patients carrying the HLA-G^*^01:04 haplotype had lower blood levels of soluble HLA-G at the time of transplantation and 3 months after transplantation (23). Interestingly, the 5-year CLAD-free rates were 44% ± 25 for patients with the HLA-G^*^01:04 haplotype and 73.4% ± 8 for patients with the other haplotypes (*p* = 0.034). Recipients with the HLA-G^*^01:04 haplotype also had an increased risk of death and produced *de novo* DSAs. In another study, HLA-G protein expression in lung biopsy was associated with a reduced risk of CLAD and anti-HLA antibodies were more frequent when HLA-G was not expressed. But serum HLA-G level was not found to predict allograft acceptance, which minor its potential as a non-invasive biomarker, despites its increase was found at 12 months post-transplantation in serum from patients with CLAD development during follow-up (25).

HLA-E has immune-tolerogenic properties, and two alleles, HLA-E^*^01:03 and HLA-E^*^01:01, have different relative peptide affinities and are involved in differences in function, cell surface expression, and potential lytic activity in NK cells. In a retrospective study performed on 119 LTRs with a mean follow-up time of 37.2 months, the HLA-E^*^01:01/01:03 heterozygous state was associated with a survival advantage at 2 years post-transplantation compared to the HLA-E^*^01:01 or HLA-E^*^01:03 homozygous state (80% for the heterozygous state vs. 50% for both homozygous states). The HLA-E^*^01:03 allele was associated with CLAD occurrence in multivariate analysis, but not with production of *de novo* DSAs (24).

### Anti-human Leucocyte Antigen (HLA) Antibody Monitoring

As reported for other solid organs, the presence of DSAs is clearly associated with increased CLAD incidence and reduced allograft survival (3, 26–30). Patients with pretransplant anti-HLA antibodies were found to have worse survival than patients without pretransplant sensitization according to a panel reactive antibody test (27, 31), and such patients also had a higher incidence of BOS (56 vs. 23%; *p* = 0.044; PRA>10%) (32).These data have been confirmed by more sensitive techniques, mainly single-bead antigen-based techniques (33, 34). Interestingly, Bosanquet et al. showed that pre-transplant allosensitization does not affect outcomes after lung transplantation when the potentially reactive HLAs are avoided in the donor by a virtual crossmatch with the recipient (35). These results have led to the refinement of organ allocation, decreasing the organ pool for sensitized patients; thus, desensitization therapies are being evaluated, with some improved patient outcomes in moderately sensitized lung recipients (36) but not in highly sensitized lung recipients (37).

After transplantation, the development of *de novo* DSAs is also clearly associated with CLAD development and/or decreased allograft survival (29, 30, 38). A recent study identified DSA, but not non-DSA anti-HLA antibodies, as risk factors for CLAD and correlates of graft survival (3). In this study, 44% of patients with DSA (persistent or transient) experience CLAD whereas only 19% of patients with no DSA develop CLAD. Koutsokera et al. found that the presence of class II *de novo* DSAs after 1 year was associated with the onset of CLAD (both BOS and RAS) at 3 years post-transplantation (39). The Toronto group identified in their cohort a 47% incidence of cumulative *de novo* DSAs, predominantly DQ-DSAs, which were strongly associated with CLAD (40). Among the abovementioned works, there is also increasing evidence for an association between the development of *de novo* DSAs and RAS. DSA measurement is now routinely performed in recipient follow-up, making it the sole biomarker used in addition to functional measures. However, the timeframe between DSA detection and CLAD diagnosis is highly variable between patients, suggesting that DSAs are an imperfect biomarker that needs improvement. DSAs are heterogeneous, with differences in antigen recognition, subclasses and the ability to bind complement. Recent reports revealed that C1q-binding DSAs can refine the stratification of DSA-bearing patients. C1q-binding DSAs detected in the first year after LT increased the risk of both CLAD and graft loss, with hazard ratios of 2.6 and 2.98, respectively, compared to that of patients without DSAs or with non-complement-fixing DSAs (41). According to these data and a report from the ISHLT, CLAD in the presence of circulating DSAs may originate from persistent antibody-mediated rejection (AMR). This “chronic” AMR could be considered as a new distinct subtype of CLAD (42). Furthermore, Magro et al. highlighted the participation of humoral immunity in BOS evolution, with notable deposits of complement molecules (C1q, C3, C4d, and C5b-9) and immunoglobulin in biopsies (43). However, compared to patients who develop transient or no DSAs (10% RAS/30% BOS and 3% RAS/16% BOS, respectively), patients with persistent DSAs who develop CLAD have been described as more likely to display RAS than BOS (20 and 27%, respectively) (*p* = 0.034) (3). These results are also consistent with the more pronounced antibody-mediated response in RAS patients than in BOS patients, as reported by this team, with more total immunoglobulins in BAL and the presence of lymphoid follicles in allografts from RAS patients (44, 45). The precise role of DSAs and humoral immunity needs to be further investigated. However, the possible involvement of B cells in RAS could result in individualized treatment to improve efficacy, and a functional approach based on DSAs would enable better risk stratification for CLAD.

## Monitoring of Auto-Immunity

In parallel of anti-HLA antibodies, antibodies against self-antigens have been evidenced as associated with DSA and/or CLAD development. Serum antibodies to collagen V and K-α 1 tubulin from 108 LTR were evaluated retrospectively in a single-center study (46). The authors reported that the development of antibodies to self-antigens was an independent risk factor for BOS. They also found that the recipients who developed DSAs were more likely to have antibodies to self-antigens. Development of DSAs could activate autoimmunity as shown in a retrospective analysis of 42 lung transplant recipients in which DSA onset preceded development of antibodies to self-antigens (47). Other studies showed self-antigens antibodies may exist before transplantation (48, 49). Tiriveedhi et al. determined that patients with pre-transplant self-antigens antibodies had higher incidence of DSA development and BOS post-transplantation (50). These data rise the hypothesis of a cross talk between immune response to HLA and self-antigens. Antibodies to self-antigens may also contribute to the pathogenesis of CLAD by activating complement (48), and/or Th17 pathway (47).

## Monitoring of Cell-Mediated Immunity

### Regulatory Cells

**Figure 2:**
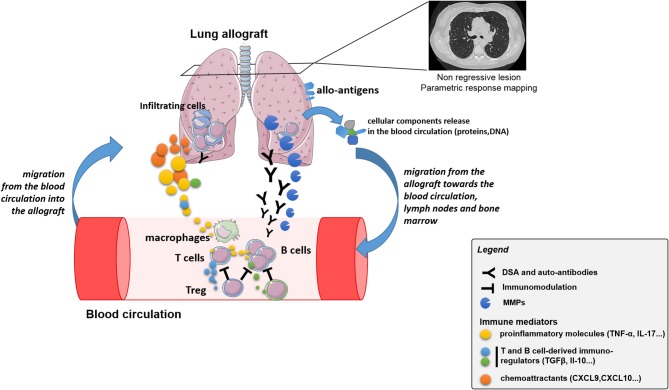
Schematic representation of the crosstalk between immune cells and allograft, with associated potential biomarkers, leading to CLAD.

In accordance with the clear involvement of the immune system in BOS biology, several reports analyzed the percentages or numbers of immune cell subsets in BALF or peripheral blood during CLAD evolution; the results are summarized in Table 1 and illustrated in Figure 2. The population that has been analyzed most thoroughly in the CLAD setting is CD4^+^ T regulatory cells (Tregs), which are key players in immune regulation. However, with respect to renal transplantation, the involvement and diagnostic usefulness of Tregs for CLAD remain unclear (60). Recent studies reported apparently conflicting results regarding the role of these cells in the context of CLAD. The Hanover group performed a prospective, single-center study with Tregs measurements from whole blood samples before transplantation and 3 weeks, 3, 6, 12, and 24 months after transplantation (2). A total of 138 patients were included in the analysis, of whom 31 (23%) developed CLAD within the 2-year follow-up. The difference in CD4^+^CD25^high^ cell frequencies between the CLAD-free and CLAD groups was not statistically significant. However, the percentages of CD127^low^, FoxP3^+^, interleukin 2 (IL-2)^+^ and CD152^+^ cells were all increased in the CLAD-free group as early as 3 weeks post-transplantation. The multivariate analysis suggested that increasing peripheral blood frequencies of CD4^+^CD25^high^CD127^low^, CD4^+^CD25^high^FoxP3^+^, and CD4^+^CD25^high^IL-2^+^ T cells 3 weeks after LT were protective factors against the early development of CLAD. Accordingly, Meloni et al. described a decrease in CD4^+^CD25^high^ Tregs numbers in peripheral blood from patients after BOS diagnosis compared to those in peripheral blood from patients with stable function (51). The same group confirmed the decrease of CD4^+^CD25^high^CD127^−^ Treg cell counts was associated with an increased risk to develop CLAD, irrespective of the RAS or BOS diagnostic, in a retrospective longitudinal analysis of 137 patients. Interestingly, the level of Treg was associated with BOS severity (52). Our group profiled CD4^+^ cells collected prospectively from patients within the longitudinal COLT study (53). Overall, 82 patients were enrolled (50 stable and 32 with BOS). The samples were analyzed at 1 and 6 months post-transplantation and at the time of BOS diagnosis. No difference in total, naive or follicular helper CD4^+^ T cell blood frequencies was observed between the BOS and stable groups, but the proportion of circulating CD4^+^CD25^high^Foxp3^+^ cells was increased at 1 and 6 months post-transplantation in BOS patients compared to that in stable patients. The Tregs compartment was further analyzed according to the expression of CD45RA, FoxP3, CD15s, and CD39; no differences were found, and Tregs from BOS and stable patients showed similar cytokine expression profiles. These results were confirmed in an independent cohort of 35 patients, in which LTRs with a higher proportion of circulating CD4^+^CD25^high^Foxp3^+^ cells at 6 months had a higher risk of developing BOS in the next 3 years. The cause for the divergent results is unclear, as it appears that the same subset of T cells was investigated, but it should be noted that in Salman's and Piloni's work, none of the LTRs received induction treatment with thymoglobulin or basiliximab, whereas more than half of our recipients received such treatment. Interestingly, Mamessier et al. also found increases in CD3^+^CD4^+^CD25^high^CD69^−^ Tregs numbers in both peripheral blood and BALF compared to those in stable LTRs, but they also observed increased levels of Tregs in BALF from BOS patients whose functional decline stopped compared to those in BALF from patients with evolving BOS (54). The evolution of BOS or the level of inflammation in the lung may explain the observed discrepancies. Tregs mobilization to downregulate lung inflammation would be consistent with results from Tiriveedhi et al., who reported that adoptive transfer of Tregs abrogated obliterative airway lesions in a mouse model after intrabronchial administration of MHC class I antibodies (61).

**Table 1:** Immune cells proposed as CLAD biomarkers.

**Cell type**	**Phenotype used**	**Compartment**	**Main observation**	**References**
Treg	CD4+CD25^high^CD127^low^, CD4+CD25^high^FoxP3^+^, CD4^+^CD25^high^IL-2^+^	Peripheral blood	Decreased level from 3 weeks after LT associated with CLAD	(2)
	CD3+CD4+CD25^high^	Peripheral blood	Decreased after BOS diagnosis	(51)
	CD4^+^CD25^high^CD127^−^	Peripheral blood	Decreased level associated with CLAD	(52)
	CD4^+^CD25^high^Foxp3^+^	Peripheral blood	Increased at 1- and 6-months post-transplantation in BOS	(53)
	CD4^+^CD25^high^CD69^−^	peripheral blood and BALF	Increased after BOS diagnosis	(54)
	CD4^+^FoxP3^+^	BALF	Decreased proportions in BALF before BOS	(55)
CD8+ lymphocytes	CD27^+^ CD45RO^+^ central memory CD8^+^ T cells	Peripheral blood	Decreased in patients developing BOS	(56)
NKT cells	CD3+CD16/56+	Peripheral blood	Increased frequencies in patients developing BOS	(56)
B lymphocytes	IgM^+^IgD^−^ memory B cells	Peripheral blood	Decreased in patients developing BOS	(56)
Total lymphocytes		BALF	Increased number in both BOS and RAS Increased frequency in RAS	(57)
		BALF	Decreased number in both BOS	(58)
		BALF	Increased frequency before CLAD	(13)
Monocytes			Decreased number in BOS	(56)
Macrophages		BALF	Decreased number in BOS	(58)
		BALF	Increased number in both BOS and RAS Decreased frequency in both BOS and RAS	(57)
Neutrophils		BALF	Increased number in BOS	(59)
		BALF	Increased number and frequency in both BOS and RAS	(57)
		BALF	Increased number and frequency in BOS	(58)
		BALF	Increased frequency before CLAD	(13)

While peripheral blood does not necessarily reflect events in the allograft, the results from BALF or biopsies performed on small cohorts are also contradictory. A study found decreased proportions of CD4^+^FoxP3^+^ cells in BALF from patients who later developed BOS but not in peripheral blood (55). Gregson et al. reported that a higher frequency of CCR7^+^CD3^+^CD4^+^CD25^high^Foxp3^+^CD45RA^−^ lymphocytes in lung allografts is associated with protection against subsequent development of BOS (62). Finally, another study found no association between the number of FoxP3^+^ cells in transbronchial biopsies and BOS development (63). While further studies are needed to ascertain the role of Tregs in CLAD evolution, such discrepancies between studies compromise the utility of these cells as a biomarker.

### Other Immune Cells

Regarding other immune cells, Budding et al. reported variations in peripheral blood samples collected monthly up to 1-year post-LT between BOS patients and stable patients (56). Overall, during the first year after LT, the authors observed decreased frequencies of total T lymphocytes and monocytes and increased frequencies of B lymphocytes. At 5 months post-LT, a timepoint selected because the patients are more stable and have reduced immunosuppression, the authors observed increased frequencies of natural killer T cells and decreased frequencies of IgM^+^IgD^−^ memory B cells and CD27^+^CD45RO^+^ central memory CD8^+^ T cells in patients developing BOS, while they observed no differences for Tregs. Despite these significant differences, the investigators failed to define a cut-off for separating BOS patients from BOS-free patients. Conversely, Durand et al. found no significant differences in the CD8 cell compartment, including CD45RA^−^CCR7^+^ central memory CD8^+^ T cells, between BOS LTRs and stable LTRs (53).

In BALF, early neutrophilia has been studied as a potential predictive biomarker. Neurohr et al. showed that neutrophilia>20% during the first year post-LT without confounding factor, was predictive for BOS with a sensitivity of 0.81 and a specificity of 0.89 (59). Siddiqui et al. also reported the positive association of BALF neutrophilia at 3 months post-transplantation with CLAD at 3 years post-transplantation in a multivariate Cox survival analysis (13). Similarly, Fisichella et al. evaluated immune mediators in prospectively collected BALF from 105 patients (64). They found elevated levels of IL-15, IL-17, tumor necrosis factor-α (TNF-α) and myeloperoxidase, highlighting the role of neutrophilic inflammation, and lower levels of alpha 1 antitrypsin in BALF collected between 6 and 12 months after transplantation from patients who developed BOS by 30 months post-transplant.

At CLAD diagnosis, Vandermeulen et al. reported an increased total cell count in BOS patients compared to that in stable patients and increased frequencies and counts of neutrophils, lymphocytes and macrophages in both BOS and RAS patients. RAS patients also displayed increased percentages and numbers of eosinophils in BALF (57). Vanaudenaerde et al. observed increased numbers of lymphocytes and neutrophils but a decreased percentage of macrophages in BOS (58).

Altogether, no immune cell has emerged as a potential biomarker for CLAD, neither in BALF nor in peripheral blood. The analysis of small cohorts with varying methodologies [whole blood vs. peripheral blood mononuclear cells (PBMCs), frozen vs. fresh cells] and CLAD stages has certainly hampered such discoveries, and this topic must be explored in larger cohorts.

## Biomarkers of Allograft Injury

Initial lung injury after transplantation has been identified as a risk factor for CLAD. Organizing pneumonia (OP) is one of the four histopathologic injury patterns of the lung allograft response to insults. OP is associated with extravasation and infiltration of mononuclear cells into the area of injury. Shino et al. sought to link OP to subsequent CLAD by analyzing levels of CXCR3 ligands (CXCL9, CXCL10, and CXCL11), which are potent chemoattractants for mononuclear cells, in BALF from LTRs (65). In this retrospective single-center study, 690 BALF samples from 324 recipients were assayed, of which 602 were from “healthy” patients and 88 were from LTRs with OP. Median CXCR3 ligand BALF concentrations were significantly higher in OP samples than in “healthy” samples. In a multivariable model adjusted for other injury patterns (acute cellular rejection, lymphocytic bronchiolitis and diffuse alveolar damage), episodes of OP with high CXCR3 ligand concentrations were associated with a significant increase in CLAD risk, in a dose-dependent manner, whereas OP episodes alone were not. This association was found for each ligand separately and when aggregated. Interestingly, when analyzing subtypes of CLAD in the same multivariable model, OP with or without CXCR3 ligands was a strong risk factor for RAS [HRs of 1.9 (95% CI 0.9–3.8) and 3.0 (95% CI 1.4–6.6), respectively] but not for BOS. This finding is one of the first breakthroughs in understanding the specific physiopathology of RAS.

CD59 was also recently identified as a potential serum biomarker for predicting BOS. CD59 is a protein attached to the cellular membrane, inhibits the membrane attack complex and is highly expressed by bronchial epithelial cells. Cell damage or activation can induce the release of the membrane-anchored protein into the circulation in a soluble form. Budding et al. performed a single-center analysis of soluble CD59 serum concentrations before transplantation and at fixed timepoints after LT in 89 patients who underwent LT (66). Patients who developed BOS (*n* = 20) had higher soluble CD59 titers than those in non-BOS patients at 6 months post-transplantation. Serum concentrations were also increased in BOS patients at the time of the clinical diagnosis of CLAD.

There is also growing interest in circulating cell-free DNA, which is likely released from cells during apoptosis. Donor-derived cell-free DNA has been shown to be relevant in early acute rejection and AMR detection (67, 68). The main characteristic of this approach is that it is not specific for CLAD but reflects lung allograft injury. In a predictive approach, this lack of specificity could be a strength since this method is able to identify allo-immune and non-alloimmune lung injury, both involved in the pathogenesis of CLAD.

New targets have also emerged recently, such as donor exosomes, which could be integrated into future CLAD prediction strategies. Exosomes are nanovesicles of endocytic origin that contain cytosolic proteins, messenger RNA and miRNA. They can be secreted by epithelial cells and are thought to be able to trigger airway inflammation by inducing macrophage activation. Moreover, MHC class I and II can be detected on exosomes and can induce specific immunity. In a case-control observational study, Gunasekaran et al. isolated exosomes from BALF and serum from LTRs with BOS, acute rejection or a “stable” condition (10 patients per condition) (69). They demonstrated that exosomes derived from LTRs contain self-antigens (collagen V) in greater proportions in patients with BOS or acute rejection than in “stable” patients, both in the BALF and in the blood. They determined by HLA analysis that the exosomes were released from the transplanted lungs after an allo-immune response, supporting the notion that both cellular and humoral rejection processes induce the formation of exosomes. In patients with BOS, exosomes containing collagen V were detected in the serum as early as 6 months post-transplantation, before clinical diagnosis. In contrast, the collagen V signal of stable LTRs decreased to undetectable levels at 3 and 6 months after transplantation. miRNA from exosomes in pooled serum samples from the same 30 patients were also investigated. Global miRNA profiling revealed high expression of miR-92a, miR-182 miR-155 and miR-142-5p in BOS patients; these miRNAs are involved in endothelial activation, inflammation, Th-17 activation and AMR, respectively. Interestingly, miR-142-5p was also found to be increased in chronic AMR after renal transplantation (70).

## Biomarkers of Airway Remodeling

Following the chronology of CLAD physiopathology, chronic inflammation is responsible for impaired wound healing and fibrotic processes. The increased activity of lung myofibroblasts and the abnormal accumulation of extracellular matrix, which are responsible for small airway remodeling, are hallmarks of BOS. Our group identified MMP-9 as a strong biomarker of epithelial-to-mesenchymal transition in an *ex vivo* model (71). Further analysis of the blood levels of MMP-9 in 94 LTRs included in the COLT study was performed every 6 months after transplantation. Plasma from patients with CLAD was compared to that from stable patients, and we found elevated levels of MMP-9 up to 12 months before the clinical diagnosis of BOS. This association was not significant for patients with RAS. The average TGF-β level was also higher in CLAD patients at all timepoints. A previous work by Kastelijn et al. reported a similar conclusion with a smaller population (72). Ramirez et al. performed an analysis of BALF retrieved at a median time of 140 days before the onset of BOS. They reported greater MMP-9 activity in BALF from 13 patients who developed BOS, as well as increased fibroblast induction of fibronectin by BALF from BOS recipients *in vitro* (73).

Another protein involved in the regulation of lung inflammation and remodeling, YKL-40, was investigated by Jaksch et al. in a population of 142 LTRs (74). They found higher pre-transplant concentrations of serum YKL-40 in patients with BOS than in patients without BOS; logistic regression analysis confirmed that YKL-40 was predictive of the development of BOS.

## Gene Expression Profiling

Gene expression analysis has been performed in several studies to identify biomarkers of CLAD. Due to the development of microarrays and high-throughput RNA sequencing, the whole transcriptome can be analyzed in a single experiment even if highly dimensional data complicate the analyses.

We recently reported the downregulation of 3 genes that can predict BOS at least 6 months before diagnosis using microarray-based gene expression profiling: POU class 2 associating factor 1 (*POU2AF1*), T cell leukemia/lymphoma protein 1A (*TCL1A*) and B cell lymphocyte kinase (*BLK*)(75). In this study, the results from a discovery cohort (49 samples from patients with stable function for at least 3 years and 32 samples collected at least 6 months before BOS diagnosis) were validated using individual qPCR in 26 independent samples. Each gene exhibited excellent area under the receiver operating characteristic (ROC) curve values (*POU2AF1*: 0.83, *TCL1A*: 0.77, and *BLK*: 0.78) and allowed the stratification of BOS risk in survival analysis. While further validation is ongoing, these results suggest the existence of a qualitative defect in B cells in patients with BOS, with an enrichment of downregulated B cell-related genes and no modification of the relative fraction of B cells.

The UCLA team performed gene expression analysis using microarrays of BALF cell pellets retrieved 1 year post-transplantation from LTR who developed CLAD within 2 years following bronchoscopy and patients who remained free of CLAD for at least 4 years (76). They identified 40 unique differentially expressed genes between CLAD and CLAD-free patients. Interestingly, these genes were related to cytotoxic lymphocyte functions, including the overexpression of genes encoding the CD8A molecule, granzymes A and H and perforin 1. A supervised machine learning analysis revealed that the top 10 features were able to correctly categorize most samples with an accuracy of 94.1%. This study was performed on 17 patients, and further confirmation is needed with a larger validation cohort. Nevertheless, these data are consistent with the increased frequencies and counts of neutrophils and lymphocytes observed in BALF from CLAD patients, suggesting the persistence of inflammation within the allograft (44, 56, 58).

A recent study measured an 11-gene signature (*CD6, TAP1, CXCL10, CXCL9, INPP5D, ISG20, LCK, NKG7, PSMB9, RUNX3*, and *BASP1*) called the common rejection module (CRM) in BALF, transbronchial brushings (TBBs) and explanted lung tissue (77). This CRM was initially identified as a diagnostic tool for acute rejection in a gene expression microarray meta-analysis of kidney, lung, heart, and liver allograft biopsies (78). At 6 months post-transplantation, CRM value was significantly higher in 4 TBBs with biopsy-proven acute rejection than in 10 TBBs without evidence of rejection, but it was not significantly different between 8 BALF samples from patients with acute rejection and 13 control samples. Regarding CLAD, the CRM in explanted tissues was significantly higher in RAS lungs (*n* = 16) than in unused donor lungs (*n* = 15), and value of a new 2-gene model (*ISG20* and *CXCL9*) was significantly lower in unused donor lungs than in RAS or BOS lungs (*n* = 13). Despite the small number of samples and the differences in time post-transplantation among the analyzed samples, this study suggests that a gene signature identified in biopsies is not applicable to different compartments, such as BALF. The usefulness of the new 2-gene score for discriminating RAS from BOS must also be highlighted in non-explanted biopsies, ideally during the early stage of CLAD, although its clinical usefulness would remain limited, as therapeutic options are not available.

Several genes associated with CLAD have been reported from additional hypothesis-driven studies. Jonigk et al. analyzed the expression of 45 tissue-remodeling and myofibroblast-related genes, which were previously identified in explanted lung allografts, in biopsy samples (79). Lung biopsies were performed within the first year post-transplantation for patients who developed CLAD (*n* = 18) in the first 3 years after transplantation and for patients without CLAD (*n* = 18) within the same observational period. The authors identified 22 differentially expressed genes. Despite the invasive nature of biopsies, the authors established a score based on 5 overexpressed genes in BOS samples, *BMP4, IL6, MMP1, SMAD1*, and *THBS1*, which correlated with CLAD clinical outcomes. In BALF from 36 BOS and 42 stable patients, Vanaudenaerde et al. highlighted the overexpression of genes encoding TGF- β, Il-17, Il-23, and Il-8, as well as Il-1B, Il-2, Il-6, and Il-8, but not TGF-β protein (58). While this result shows the involvement of proinflammatory Th17 cells in BOS mechanisms, the diagnostic properties of these markers were not evaluated. The increase in most of these molecules in BALF from patients experiencing infections suggests that they are related to inflammation and are not specific to BOS.

In addition to protein-expressing genes, non-coding RNAs can also be profiled and used as biomarkers. Notably, microRNAs (miRNAs), which are short non-coding RNAs that modulate gene expression by binding messenger RNA, are relatively resistant to degradation and can be found in various biological fluids, including serum. Budding et al. compared serum levels of profibrotic (miR-21 and miR-155), antifibrotic (miR-29a), and fibrosis-unrelated (miR-103 and miR-191) miRNAs during LT follow-up between 10 patients who developed BOS and 10 patients who did not (80). Blood samples were available at four timepoints between 9 and 38 months after transplantation. The levels of pro- and antifibrotic miRNAs were elevated in serum from BOS patients prior to the diagnosis of BOS, as early as 9 months post-transplantation.

Altogether, these profiling studies identified genes with altered expression in biopsies, BALF and peripheral whole blood from patients who later developed CLAD. While these studies contributed to the identification of CLAD mechanisms, including fibrosis, and remodeling processes, few of them provided potential robust biomarkers of CLAD. Moreover, challenges of such studies remain the use of larger cohorts of patients and proper external validation.

## Conclusion

The search for biomarkers has not only enabled the emergence of possible predictive or diagnostic tools but also proved to be a relevant approach for improving our understanding of the post-transplantation process. Consequently, knowledge on the mechanisms of CLAD and BOS and RAS patterns has greatly increased over the past decade. It is reasonable to postulate that new phenotypes will emerge in the coming years, and it is also likely that different mechanisms will be identified, leading to specific subtypes of CLAD. All the works described in this review are promising and demonstrate the strong commitment of the clinical and scientific community to improving LT outcomes. However, the multiple presentations of CLAD and the relatively small number of lung transplants compared to other solid organ transplants make elucidating this problem complex and burdensome. From this review, two conclusions can be drawn. (i) There is a need for validation of potential biomarkers on a larger scale, and collaboration between centers is therefore needed to reach a sufficient population size. Some consortia have recently emerged in Europe or in North America, with the aim of developing collaborative research in lung transplantation. Validation of biomarkers should be necessarily leaned on these groups to elevate the quality of research at a upper level, (ii) As the ideal biomarker probably does not exist, several combined biomarkers will be needed for follow-up and risk stratification at the individual level to encompass several presentations of CLAD. These combinations of predictive markers will have to take in account risk factors that may be important for stratification despites not being predictive *per se*. This is the case for environmental factors, including air pollution (81), smoking experience (82) and also non-observance (83), a possible under-estimated risk of CLAD. Furthermore, step by step processes using biomarkers would need to be designed, starting from allograft matching, then with screening biomarkers that could be used to monitor patients regularly. These parameters would be highly sensible, indicating the need for more precise and specific ones leading to accurate lung allograft dysfunction diagnosis according to the current state of art. Precision medicine and different tools associated with this approach may be applicable in this context, in which biomarkers, internal (demographic, genetic, and underlying disease) and external (pollution, infection episodes, etc.) risk factors should be integrated into a clinical decision support system (CDSS) that can be used by clinicians and patients, with the potential to adapt preventive and, in the future, curative strategies.

## Author Contributions

AT, RD, AM, and SB contributed to the conception and design of the review. AT and RD wrote the first draft of the manuscript. JC, AM, and SB revised the manuscript critically and added important intellectual content. All authors contributed to manuscript revision, read, and approved the submitted version.

### Conflict of Interest Statement

The authors declare that the research was conducted in the absence of any commercial or financial relationships that could be construed as a potential conflict of interest.

## Abbreviations

AMR: antibody-mediated rejection
BALF: bronchoalveolar lavage fluid
BOS: bronchiolitis obliterans syndrome
CDSS: clinical decision support system
CLAD: chronic lung allograft dysfunction
CRM: common rejection module
CT scan: computed tomography scanner
DSA: donor specific antibody
FEV1: forced expiratory volume in 1 second
HLA: human leucocyte antigen
ISHLT: International Society for Heart and Lung Transplantation
LT: lung transplantation
LTR: lung transplant recipient
miRNA: microRNA
MMP: matrix metalloproteinase
OP: organizing pneumonia
PGD: primary graft dysfunction
PRM: parametric response mapping
RAS: restrictive allograft syndrome
TBB: transbronchial brushings
Treg: T regulatory cell
4P medicine, predictive, preventive: personalized and participatory medicine.
